# 1-{[3-(2-Chloro-3,3,3-trifluoro­prop-1-en­yl)-2,2-dimethyl­cyclo­propan-1-yl]carbon­yl}-3-(methyl­sulfon­yl)imidazolidin-2-one

**DOI:** 10.1107/S1600536812021216

**Published:** 2012-05-16

**Authors:** Na-Bo Sun, Guo-Wu Rao, Jian-Bo Chu

**Affiliations:** a College of Biology and Environmental Engineering, Zhejiang Shuren University, Hangzhou 310015, People’s Republic of China; b College of Pharmaceutical Science, Zhejiang University of Technology, Hangzhou 310014, People’s Republic of China; c Department of Pharmacy, Zhejiang Medical College, Hangzhou 310053, People’s Republic of China

## Abstract

In the title mol­ecule, C_13_H_16_ClF_3_N_2_O_4_S, the imidazolidine ring is approximately planar, the largest deviation from this plane being 0.025 (3) Å. The cyclo­propane ring forms a dihedral angle of 64.1 (2)° with the imidazolidine ring. In the crystal, C—H⋯O hydrogen bonds are observed.

## Related literature

For the biological activities of pyrethroids, see: Chen & Yu (1991 ▶); Sun *et al.* (2008 ▶). For the crystal structures of similar compounds, see: Sun, Shen, Rao *et al.* (2006 ▶); Sun, Shen, Zheng *et al.* (2006 ▶). For the synthesis of the title compound, see: Sun *et al.* (2008 ▶).
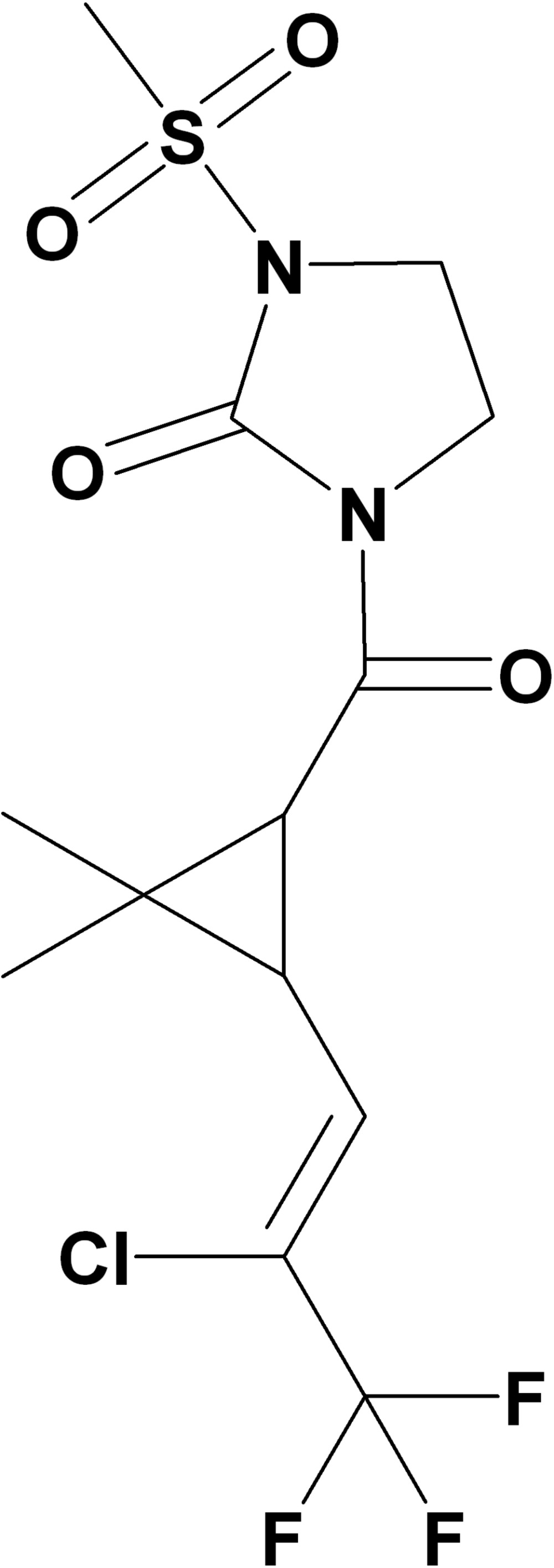


## Experimental

### 

#### Crystal data

C_13_H_16_ClF_3_N_2_O_4_S
*M* *_r_* = 388.79
Monoclinic, 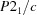

*a* = 15.404 (4) Å
*b* = 9.483 (2) Å
*c* = 11.858 (3) Å
β = 103.464 (4)°
*V* = 1684.5 (8) Å^3^
*Z* = 4
Mo *K*α radiation
μ = 0.40 mm^−1^
*T* = 298 K
0.68 × 0.40 × 0.18 mm

#### Data collection

Bruker SMART CCD diffractometer
Absorption correction: multi-scan (*SADABS*; Bruker, 1997 ▶) *T*_min_ = 0.761, *T*_max_ = 0.930
6863 measured reflections
2964 independent reflections
2548 reflections with *I* > 2σ(*I*)
*R*_int_ = 0.022

#### Refinement

*R*[*F* ^2^ > 2σ(*F* ^2^)] = 0.062
*wR*(*F* ^2^) = 0.175
*S* = 1.11
2964 reflections
217 parameters
H-atom parameters constrained
Δρ_max_ = 0.57 e Å^−3^
Δρ_min_ = −0.53 e Å^−3^

### 

Data collection: *SMART* (Bruker, 1997 ▶); cell refinement: *SAINT* (Bruker, 1997 ▶); data reduction: *SAINT*; program(s) used to solve structure: *SHELXS97* (Sheldrick, 2008 ▶); program(s) used to refine structure: *SHELXL97* (Sheldrick, 2008 ▶); molecular graphics: *ORTEP-3 for Windows* (Farrugia, 1997 ▶); software used to prepare material for publication: *WinGX* (Farrugia, 1999 ▶).

## Supplementary Material

Crystal structure: contains datablock(s) I, global. DOI: 10.1107/S1600536812021216/wn2475sup1.cif

Structure factors: contains datablock(s) I. DOI: 10.1107/S1600536812021216/wn2475Isup2.hkl

Supplementary material file. DOI: 10.1107/S1600536812021216/wn2475Isup3.cdx

Supplementary material file. DOI: 10.1107/S1600536812021216/wn2475Isup4.cml

Additional supplementary materials:  crystallographic information; 3D view; checkCIF report

## Figures and Tables

**Table 1:** Hydrogen-bond geometry (Å, °)

*D*—H⋯*A*	*D*—H	H⋯*A*	*D*⋯*A*	*D*—H⋯*A*
C2—H2*A*⋯O4^i^	0.97	2.50	3.267 (5)	136
C3—H3*A*⋯O4^ii^	0.97	2.50	3.352 (6)	146
C13—H13*C*⋯O2^iii^	0.96	2.57	3.331 (6)	137

Symmetry codes: (i) 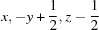; (ii) 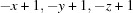; (iii) 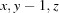.

**Fig. 1.**
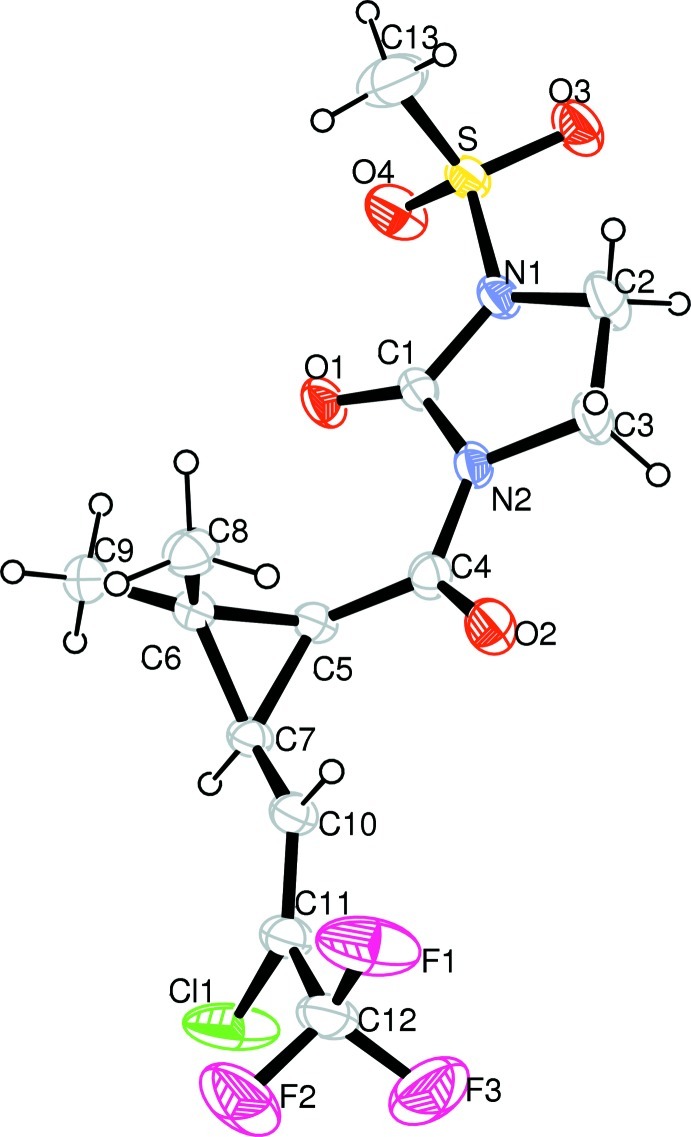
The molecular structure of the title compound, shown with 30% probability displacement ellipsoids. Hydrogen atoms are shown as spheres of arbitrary radius.

**Fig. 2.**
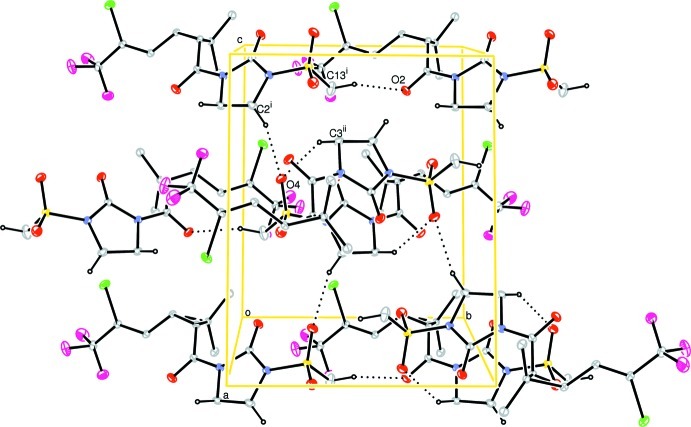
A portion of the crystal packing of the title compound. Hydrogen bonds are shown as dashed lines. H atoms not involved in hydrogen bonding have been omitted for clarity.

## supplementary crystallographic information

### Comment

Pyrethroids have a high potential for biological activity with low toxicity and good environmental compatibility. They have been widely used in making pesticides (Chen & Yu, 1991; Sun *et al.*, 2008). In a continuation of our studies of the biological activities of pyrethroids, we have obtained a colourless crystalline compound, whose structure has been confirmed by single-crystal X-ray diffraction. The crystal structures of two similar compounds have already been published (Sun, Shen, Rao *et al.*, 2006; Sun, Shen, Zheng *et al.*, 2006).

The molecular structure of the title compound is illustrated in Fig. 1. Atoms N1, C2, C3, N2 and C1 are approximately planar, the largest deviation from this plane being 0.025 (3) Å for atom N1. The cyclopropane ring (C5—C7) forms dihedral angles of 89.49 (22) ° and 64.07 (21) ° with the least-squares planes of the C8, C9, C6 grouping and the imidazolidine ring, respectively. In the crystal structure, intermolecular C—H···O hydrogen bonds are observed. A short intermolecular contact of O3···C3 = 3.02 Å is present.

### Experimental

The title compound was synthesized according to the published procedure (Sun *et al.*, 2008). A solution of the compound in ethanol was concentrated gradually at room temperature to afford colourless blocks.

### Refinement

H atoms were included in calculated positions and refined using a riding model. Csp^2^—H = 0.93 Å, Cmethyl—H = 0.96 Å, Cmethylene—H = 0.97 Å and Cmethine—H = 0.98 Å. U_iso_(H) = xU_eq_(C), where x = 1.5 for methyl H and 1.2 for all other H atoms.

### Crystal data

**Table:**

C_13_H_16_ClF_3_N_2_O_4_S	*F*(000) = 800
*M**_r_* = 388.79	*D*_x_ = 1.533 Mg m^−^^3^
Monoclinic, *P*2_1_/*c*	Mo *K*α radiation, λ = 0.71073 Å
Hall symbol: -P 2ybc	Cell parameters from 3411 reflections
*a* = 15.404 (4) Å	θ = 2.7–26.7°
*b* = 9.483 (2) Å	µ = 0.40 mm^−^^1^
*c* = 11.858 (3) Å	*T* = 298 K
β = 103.464 (4)°	Block, colourless
*V* = 1684.5 (8) Å^3^	0.68 × 0.40 × 0.18 mm
*Z* = 4	

### Data collection

**Table:**

Bruker SMART CCD diffractometer	2964 independent reflections
Radiation source: fine-focus sealed tube	2548 reflections with *I* > 2σ(*I*)
Graphite monochromator	*R*_int_ = 0.022
φ and ω scans	θ_max_ = 25.0°, θ_min_ = 1.4°
Absorption correction: multi-scan (*SADABS*; Bruker, 1997)	*h* = −18→14
*T*_min_ = 0.761, *T*_max_ = 0.930	*k* = −8→11
6863 measured reflections	*l* = −14→14

### Refinement

**Table:**

Refinement on *F*^2^	Primary atom site location: structure-invariant direct methods
Least-squares matrix: full	Secondary atom site location: difference Fourier map
*R*[*F*^2^ > 2σ(*F*^2^)] = 0.062	Hydrogen site location: inferred from neighbouring sites
*wR*(*F*^2^) = 0.175	H-atom parameters constrained
*S* = 1.11	*w* = 1/[σ^2^(*F*_o_^2^) + (0.0729*P*)^2^ + 2.4194*P*] where *P* = (*F*_o_^2^ + 2*F*_c_^2^)/3
2964 reflections	(Δ/σ)_max_ < 0.001
217 parameters	Δρ_max_ = 0.57 e Å^−^^3^
0 restraints	Δρ_min_ = −0.53 e Å^−^^3^

### Special details

**Table:**

Geometry. All e.s.d.'s (except the e.s.d. in the dihedral angle between two l.s. planes) are estimated using the full covariance matrix. The cell e.s.d.'s are taken into account individually in the estimation of e.s.d.'s in distances, angles and torsion angles; correlations between e.s.d.'s in cell parameters are only used when they are defined by crystal symmetry. An approximate (isotropic) treatment of cell e.s.d.'s is used for estimating e.s.d.'s involving l.s. planes.
Refinement. Refinement of *F*^2^ against ALL reflections. The weighted *R*-factor *wR* and goodness of fit *S* are based on *F*^2^, conventional *R*-factors *R* are based on *F*, with *F* set to zero for negative *F*^2^. The threshold expression of *F*^2^ > σ(*F*^2^) is used only for calculating *R*-factors(gt) *etc*. and is not relevant to the choice of reflections for refinement. *R*-factors based on *F*^2^ are statistically about twice as large as those based on *F*, and *R*- factors based on ALL data will be even larger.

### Fractional atomic coordinates and isotropic or equivalent isotropic displacement parameters (Å^2^)

**Table:**

	*x*	*y*	*z*	*U*_iso_*/*U*_eq_	
F1	0.0849 (3)	1.1070 (3)	0.3133 (2)	0.1095 (13)	
F2	0.0338 (3)	1.2094 (4)	0.4425 (3)	0.1146 (13)	
F3	0.1607 (3)	1.2571 (3)	0.4218 (4)	0.1126 (12)	
O3	0.5270 (2)	0.1855 (3)	0.3889 (3)	0.0683 (9)	
O4	0.4640 (3)	0.1833 (3)	0.5579 (3)	0.0778 (10)	
C13	0.3639 (4)	0.1076 (5)	0.3604 (6)	0.0918 (18)	
H13A	0.3575	0.1240	0.2790	0.138*	
H13B	0.3101	0.1352	0.3822	0.138*	
H13C	0.3749	0.0092	0.3768	0.138*	
S	0.45301 (6)	0.20590 (10)	0.43865 (8)	0.0461 (3)	
Cl1	0.16342 (14)	1.09317 (14)	0.64639 (11)	0.1095 (7)	
O1	0.3400 (2)	0.4131 (3)	0.5463 (2)	0.0605 (8)	
O2	0.2822 (2)	0.7813 (3)	0.3499 (2)	0.0585 (7)	
N2	0.34790 (19)	0.5737 (3)	0.4009 (2)	0.0407 (7)	
N1	0.4231 (2)	0.3728 (3)	0.4092 (3)	0.0450 (7)	
C7	0.1933 (2)	0.7923 (3)	0.5495 (3)	0.0385 (7)	
H7	0.1997	0.8140	0.6319	0.046*	
C10	0.1603 (2)	0.9097 (4)	0.4727 (3)	0.0418 (8)	
H10	0.1507	0.8925	0.3935	0.050*	
C6	0.1633 (2)	0.6421 (4)	0.5170 (3)	0.0399 (8)	
C4	0.2957 (2)	0.6873 (4)	0.4208 (3)	0.0414 (8)	
C1	0.3662 (2)	0.4485 (4)	0.4631 (3)	0.0396 (8)	
C8	0.1030 (3)	0.6147 (4)	0.3983 (3)	0.0543 (10)	
H8A	0.1197	0.6759	0.3424	0.081*	
H8B	0.0421	0.6326	0.4007	0.081*	
H8C	0.1091	0.5183	0.3767	0.081*	
C5	0.2599 (2)	0.6822 (3)	0.5266 (3)	0.0378 (7)	
H5	0.3018	0.6469	0.5961	0.045*	
C12	0.1041 (3)	1.1499 (4)	0.4206 (4)	0.0608 (11)	
C3	0.3890 (3)	0.5794 (4)	0.3006 (3)	0.0505 (9)	
H3A	0.4298	0.6584	0.3072	0.061*	
H3B	0.3437	0.5882	0.2288	0.061*	
C11	0.1428 (3)	1.0371 (4)	0.5035 (3)	0.0492 (9)	
C2	0.4380 (3)	0.4417 (5)	0.3047 (4)	0.0604 (11)	
H2A	0.4140	0.3856	0.2362	0.072*	
H2B	0.5012	0.4573	0.3107	0.072*	
C9	0.1443 (3)	0.5501 (4)	0.6126 (4)	0.0527 (9)	
H9A	0.0815	0.5302	0.5973	0.079*	
H9B	0.1624	0.5983	0.6855	0.079*	
H9C	0.1768	0.4633	0.6158	0.079*	

### Atomic displacement parameters (Å^2^)

**Table:**

	*U*^11^	*U*^22^	*U*^33^	*U*^12^	*U*^13^	*U*^23^
F1	0.183 (4)	0.078 (2)	0.0565 (17)	0.059 (2)	0.0057 (19)	0.0025 (14)
F2	0.117 (3)	0.110 (3)	0.131 (3)	0.074 (2)	0.059 (2)	0.031 (2)
F3	0.130 (3)	0.0571 (18)	0.151 (3)	0.0027 (18)	0.032 (2)	0.032 (2)
O3	0.0670 (19)	0.070 (2)	0.078 (2)	0.0244 (15)	0.0374 (16)	0.0059 (15)
O4	0.119 (3)	0.067 (2)	0.0551 (18)	0.0320 (19)	0.0358 (18)	0.0181 (15)
C13	0.081 (4)	0.057 (3)	0.132 (5)	−0.011 (3)	0.014 (3)	−0.020 (3)
S	0.0538 (6)	0.0429 (5)	0.0461 (5)	0.0096 (4)	0.0209 (4)	0.0010 (4)
Cl1	0.2056 (19)	0.0635 (8)	0.0556 (8)	0.0415 (9)	0.0228 (9)	−0.0150 (6)
O1	0.087 (2)	0.0494 (15)	0.0611 (17)	0.0175 (14)	0.0496 (16)	0.0161 (13)
O2	0.0729 (18)	0.0535 (16)	0.0566 (16)	0.0195 (13)	0.0301 (14)	0.0212 (13)
N2	0.0455 (15)	0.0409 (16)	0.0417 (16)	0.0047 (12)	0.0223 (13)	0.0068 (12)
N1	0.0596 (18)	0.0421 (16)	0.0403 (16)	0.0084 (14)	0.0258 (14)	0.0034 (13)
C7	0.0461 (18)	0.0357 (17)	0.0349 (17)	0.0016 (14)	0.0119 (14)	−0.0058 (14)
C10	0.0476 (19)	0.0396 (18)	0.0400 (18)	0.0045 (15)	0.0137 (15)	−0.0037 (15)
C6	0.0441 (18)	0.0354 (17)	0.0434 (19)	0.0008 (14)	0.0162 (15)	−0.0036 (14)
C4	0.0396 (18)	0.0410 (19)	0.0446 (19)	0.0029 (14)	0.0118 (15)	0.0066 (15)
C1	0.0443 (18)	0.0407 (18)	0.0377 (18)	0.0016 (14)	0.0175 (14)	−0.0009 (14)
C8	0.056 (2)	0.050 (2)	0.055 (2)	−0.0069 (18)	0.0086 (18)	−0.0113 (18)
C5	0.0428 (18)	0.0356 (17)	0.0359 (17)	0.0056 (14)	0.0107 (14)	−0.0008 (13)
C12	0.075 (3)	0.045 (2)	0.066 (3)	0.019 (2)	0.024 (2)	−0.002 (2)
C3	0.061 (2)	0.057 (2)	0.043 (2)	0.0081 (18)	0.0295 (18)	0.0089 (17)
C11	0.062 (2)	0.041 (2)	0.048 (2)	0.0057 (17)	0.0191 (18)	−0.0066 (16)
C2	0.072 (3)	0.069 (3)	0.050 (2)	0.023 (2)	0.036 (2)	0.018 (2)
C9	0.061 (2)	0.047 (2)	0.057 (2)	−0.0048 (18)	0.0284 (19)	−0.0010 (18)

### Geometric parameters (Å, º)

**Table:**

F1—C12	1.303 (5)	C7—H7	0.9800
F2—C12	1.300 (5)	C10—C11	1.308 (5)
F3—C12	1.337 (6)	C10—H10	0.9300
O3—S	1.414 (3)	C6—C9	1.512 (5)
O4—S	1.401 (3)	C6—C5	1.515 (5)
C13—S	1.738 (5)	C6—C8	1.517 (5)
C13—H13A	0.9600	C4—C5	1.484 (5)
C13—H13B	0.9600	C8—H8A	0.9600
C13—H13C	0.9600	C8—H8B	0.9600
S—N1	1.663 (3)	C8—H8C	0.9600
Cl1—C11	1.734 (4)	C5—H5	0.9800
O1—C1	1.197 (4)	C12—C11	1.481 (6)
O2—C4	1.210 (4)	C3—C2	1.503 (6)
N2—C1	1.392 (4)	C3—H3A	0.9700
N2—C4	1.397 (4)	C3—H3B	0.9700
N2—C3	1.472 (4)	C2—H2A	0.9700
N1—C1	1.397 (4)	C2—H2B	0.9700
N1—C2	1.465 (5)	C9—H9A	0.9600
C7—C10	1.453 (5)	C9—H9B	0.9600
C7—C6	1.520 (5)	C9—H9C	0.9600
C7—C5	1.532 (4)		
			
S—C13—H13A	109.5	C6—C8—H8A	109.5
S—C13—H13B	109.5	C6—C8—H8B	109.5
H13A—C13—H13B	109.5	H8A—C8—H8B	109.5
S—C13—H13C	109.5	C6—C8—H8C	109.5
H13A—C13—H13C	109.5	H8A—C8—H8C	109.5
H13B—C13—H13C	109.5	H8B—C8—H8C	109.5
O4—S—O3	118.7 (2)	C4—C5—C6	119.8 (3)
O4—S—N1	108.67 (17)	C4—C5—C7	121.6 (3)
O3—S—N1	104.69 (17)	C6—C5—C7	59.8 (2)
O4—S—C13	110.6 (3)	C4—C5—H5	114.9
O3—S—C13	108.5 (3)	C6—C5—H5	114.9
N1—S—C13	104.6 (2)	C7—C5—H5	114.9
C1—N2—C4	128.4 (3)	F2—C12—F1	108.7 (4)
C1—N2—C3	112.8 (3)	F2—C12—F3	103.9 (4)
C4—N2—C3	118.8 (3)	F1—C12—F3	104.3 (4)
C1—N1—C2	113.3 (3)	F2—C12—C11	114.0 (4)
C1—N1—S	124.4 (2)	F1—C12—C11	112.8 (3)
C2—N1—S	120.8 (2)	F3—C12—C11	112.4 (4)
C10—C7—C6	121.2 (3)	N2—C3—C2	104.4 (3)
C10—C7—C5	124.2 (3)	N2—C3—H3A	110.9
C6—C7—C5	59.5 (2)	C2—C3—H3A	110.9
C10—C7—H7	113.8	N2—C3—H3B	110.9
C6—C7—H7	113.8	C2—C3—H3B	110.9
C5—C7—H7	113.8	H3A—C3—H3B	108.9
C11—C10—C7	126.7 (3)	C10—C11—C12	124.0 (4)
C11—C10—H10	116.7	C10—C11—Cl1	123.6 (3)
C7—C10—H10	116.7	C12—C11—Cl1	112.4 (3)
C9—C6—C5	116.5 (3)	N1—C2—C3	103.8 (3)
C9—C6—C8	114.4 (3)	N1—C2—H2A	111.0
C5—C6—C8	119.4 (3)	C3—C2—H2A	111.0
C9—C6—C7	116.8 (3)	N1—C2—H2B	111.0
C5—C6—C7	60.6 (2)	C3—C2—H2B	111.0
C8—C6—C7	118.7 (3)	H2A—C2—H2B	109.0
O2—C4—N2	117.5 (3)	C6—C9—H9A	109.5
O2—C4—C5	125.1 (3)	C6—C9—H9B	109.5
N2—C4—C5	117.4 (3)	H9A—C9—H9B	109.5
O1—C1—N2	127.6 (3)	C6—C9—H9C	109.5
O1—C1—N1	126.9 (3)	H9A—C9—H9C	109.5
N2—C1—N1	105.5 (3)	H9B—C9—H9C	109.5
			
O4—S—N1—C1	−37.1 (4)	O2—C4—C5—C6	77.1 (5)
O3—S—N1—C1	−164.9 (3)	N2—C4—C5—C6	−102.0 (4)
C13—S—N1—C1	81.1 (4)	O2—C4—C5—C7	6.2 (5)
O4—S—N1—C2	157.4 (3)	N2—C4—C5—C7	−172.8 (3)
O3—S—N1—C2	29.6 (4)	C9—C6—C5—C4	141.2 (3)
C13—S—N1—C2	−84.4 (4)	C8—C6—C5—C4	−3.0 (5)
C6—C7—C10—C11	144.2 (4)	C7—C6—C5—C4	−111.4 (3)
C5—C7—C10—C11	−143.6 (4)	C9—C6—C5—C7	−107.3 (3)
C10—C7—C6—C9	−139.2 (3)	C8—C6—C5—C7	108.4 (3)
C5—C7—C6—C9	106.9 (3)	C10—C7—C5—C4	−0.6 (5)
C10—C7—C6—C5	113.9 (4)	C6—C7—C5—C4	108.5 (4)
C10—C7—C6—C8	4.4 (5)	C10—C7—C5—C6	−109.1 (4)
C5—C7—C6—C8	−109.5 (3)	C1—N2—C3—C2	−0.5 (4)
C1—N2—C4—O2	−173.8 (3)	C4—N2—C3—C2	−179.1 (3)
C3—N2—C4—O2	4.6 (5)	C7—C10—C11—C12	−176.2 (4)
C1—N2—C4—C5	5.4 (5)	C7—C10—C11—Cl1	4.5 (6)
C3—N2—C4—C5	−176.3 (3)	F2—C12—C11—C10	127.9 (5)
C4—N2—C1—O1	0.5 (6)	F1—C12—C11—C10	3.4 (6)
C3—N2—C1—O1	−177.9 (4)	F3—C12—C11—C10	−114.2 (5)
C4—N2—C1—N1	−178.6 (3)	F2—C12—C11—Cl1	−52.7 (5)
C3—N2—C1—N1	3.0 (4)	F1—C12—C11—Cl1	−177.3 (4)
C2—N1—C1—O1	176.4 (4)	F3—C12—C11—Cl1	65.1 (4)
S—N1—C1—O1	10.0 (6)	C1—N1—C2—C3	4.0 (5)
C2—N1—C1—N2	−4.4 (4)	S—N1—C2—C3	171.0 (3)
S—N1—C1—N2	−170.8 (2)	N2—C3—C2—N1	−2.0 (4)

### Hydrogen-bond geometry (Å, º)

**Table:**

*D*—H···*A*	*D*—H	H···*A*	*D*···*A*	*D*—H···*A*
C2—H2*A*···O4^i^	0.97	2.50	3.267 (5)	136
C3—H3*A*···O4^ii^	0.97	2.50	3.352 (6)	146
C13—H13*C*···O2^iii^	0.96	2.57	3.331 (6)	137

Symmetry codes: (i) *x*, −*y*+1/2, *z*−1/2; (ii) −*x*+1, −*y*+1, −*z*+1; (iii) *x*, *y*−1, *z*.
